# New Coleoptera records from New Brunswick, Canada: Elateridae

**DOI:** 10.3897/zookeys.179.2603

**Published:** 2012-04-04

**Authors:** Reginald P. Webster, Jon D. Sweeney, Ian DeMerchant

**Affiliations:** 1 Natural Resources Canada, Canadian Forest Service - Atlantic Forestry Centre, 1350 Regent St., P.O. Box 4000, Fredericton, NB, Canada E3B 5P7

**Keywords:** Elateridae, new records, Canada, New Brunswick

## Abstract

Twenty-two species of Elateridae are newly reported for New Brunswick, Canada. *Negastrius exiguus* (Randall) is removed from the faunal list and *Agriotes pubescens* Melsheimer is re-instated as a member of the New Brunswick fauna. *Agriotes pubescens* Melsheimer, *Dalopius brevicornis* W. J. Brown, *Danosoma obtectum* (Say) and *Megapenthes solitarius* Fall are newly reported for the Maritime provinces. Collection data, bionomic data, and distribution maps are presented for all these species.

## Introduction

The Elateridae (click beetles) is a species-rich family of beetles with about 965 named species in North America (Johnson 2002) and 369 species and subspecies from Canada and Alaska (Bousquet 1991). Although some groups are fairly well known taxonomically, genera such as *Ampedus* and *Dalopius* are in need of revisionary study and include a number of undescribed species (Johnson 2002). Larvae of some species of Elateridae are rhizophagus and are important agricultural pests; larvae of other species are predaceous, often living in soil, subcortical habitats, or rotten logs (Johnson 2002). However, little is known about the biology of most species.

The Elateridae of the Maritime provinces (New Brunswick, Nova Scotia, Prince Edward Island) was reviewed by Majka and Johnson (2008). They provided a detailed historical overview the collection of the Elateridae and other families of beetles in the Maritime provinces and a taxonomic review of the genus *Ctenicera*, which was in need of taxonomic review and generic re-assignment. Ninety-eight species were reported for New Brunswick, 13 as new provincial records; *Agriotes pubescens* Melsheimer, *Athous campyloides* Newman, and *Cardiophorus cardisce* (Say) were removed from the faunal list of the province by Majka and Johnson (2008). Later, Douglas (2011) newly reported *Pseudanstirus nigricollis* (Bland) and the adventive *Hemicrepidius niger* (Linnaeus) from New Brunswick. *Hemicrepidius niger* was also reported from Ontario and these represented the first records of this Eurasian species from North America. Here, we newly report 22 elaterid species from New Brunswick.

## Methods and conventions

The following records are based on specimens collected during a general survey by the first author to document the Coleoptera fauna of New Brunswick and from by-catch samples obtained during a study to develop a general attractant for the detection of invasive species of Cerambycidae. Additional provincial records were obtained from specimens contained in the collection belonging to Natural Resources Canada, Canadian Forest Service - Atlantic Forestry Centre, Fredericton, New Brunswick.

### Collection methods

Various methods were employed to collect the species reported in this study. Details are outlined in Webster et al. (2009, Appendix). Many specimens were also collected from 12-unit Lindgren funnel traps set in various forest habitats in New Brunswick between 2008 and 2011. These traps mimic tree trunks and are often effective for sampling species of Coleoptera that live in microhabitats associated with standing trees (Lindgren 1983). See Webster et al. (in press) for details of the methods used to deploy Lindgren funnel traps and for sample collection. A description of the habitat was recorded for all specimens collected during this survey. Locality and habitat data are presented exactly as on labels for each record. This information, as well as additional collecting notes, is summarized and discussed in the collection and habitat data section for each species.

### Specimen Preparation

Males of some species of Elateridae were dissected to confirm their identity. The genital structures were dehydrated in absolute alcohol and mounted in Canada balsam on celluloid microslides and pinned with the specimens they originated from.

### Distribution

Distribution maps, created using ArcMap and ArcGIS, are presented for each species in New Brunswick. Every species is cited with current distribution in Canada and Alaska, using abbreviations for the state, provinces, and territories. New records for New Brunswick are indicated in bold under Distribution in Canada and Alaska. The following abbreviations are used in the text:

Acronyms of collections examined or where specimens reside referred to in this study are as follows:

**Table:** 

**AK**	Alaska	**MB**	Manitoba
**YT**	Yukon Territory	**ON**	Ontario
**NT**	Northwest Territories	**QC**	Quebec
**NU**	Nunavut	**NB**	New Brunswick
**BC**	British Columbia	**PE**	Prince Edward Island
**AB**	Alberta	**NS**	Nova Scotia
**SK**	Saskatchewan	**NF & LB**	Newfoundland and Labrador

AFCAtlantic Forestry Centre, Natural Resources Canada, Canadian Forest Service, Fredericton, New Brunswick, Canada


CNCCanadian National Collection of Insects, Arachnids and Nematodes, Agriculture and Agri-Food Canada, Ottawa, Ontario, Canada


NBMNew Brunswick Museum, Saint John, New Brunswick, Canada


RWCReginald Webster Collection, Charters Settlement, New Brunswick, Canada


## Results

Twenty-two species of Elateridae are newly reported for New Brunswick, *Negastrius exiguus* (Randall) is removed from the faunal list, and *Agriotes pubescens* Melsheimer reinstated as a member of the New Brunswick fauna, bringing the total number of species known from the province to 122. *Agriotes pubescens* Melsheimer, *Dalopius brevicornis*, *Danosoma obtectum* (Say), and *Megapenthes solitarius* Fall are newly reported for the Maritime provinces. Several apparently undescribed *Ampedus* sp. have also been found in New Brunswick but these are not reported here.

**Table 1.** Species of Elateridae recorded from New Brunswick, Canada.

**Family Elateridae Leach**
**Subfamily Agrypninae Candèze**
**Tribe Agrypnini Candèze**
*Danosoma brevicornis* (LeConte)
*Danosoma obtectum* (Say)**
*Lacon auroratus* (Say)
**Subfamily Lissominae Laporte**
*Oestodes tenuicollis* (Randall)
**Subfamily Pityobiinae Hyslop**
*Pityobius anguinius* LeConte
**Subfamily Dendrometrinae Gistel**
**Tribe Dendrometrini Gistel**
*Athous acanthus* (Say)
*Athous brightwelli* (Kirby)
*Athous fossularis* (LeConte)
*Athous orvus* Becker
*Athous posticus* (Melsheimer)*
*Athous productus* (Randall)
*Athous rufifrons* (Randall)
*Athous scapularis* (Say)*
*Denticollis denticornis* (Kirby)
*Elathous discalceatus* (Say)*
*Hemicrepidius brevicollis* (Candèze)
*Hemicrepidius hemipodus *(Say)
*Hemicrepidius memnonius *(Herbst)*
*Hemicrepidius niger* (Linnaeus)
*Limonius aeger* LeConte
*Limonius anceps* LeConte
*Limonius confusus* LeConte
*Limonius pectoralis* LeConte
**Tribe Prosternini Gistel**
*Actenicerus cuprascens* (LeConte)
*Anostirus vernalis* (Hentz)
*Beckerus appressus* (Randall)
*Corymbitodes elongaticollis* (Hamilton)
*Corymbitodes pygmaeus *(Van Dyke)
*Corymbitodes tarsalis *(Melsheimer)
*Ctenicera kendalli* (Kirby)
*Eanus estriatus* (LeConte)
*Eanus maculipennis* LeConte
*Hypoganus sulcicollis* (Say)*
*Hypoganus rotundicollis* (Say)**
*Liotrichus falsificus* (LeConte)
*Liotrichus spinosus* (LeConte)
*Liotrichus vulneratus* (LeConte)
*Metanomus insidiosus* (LeConte)
*Nitidolimonius resplendens* (Eschscholtz)
*Oxygonus montanus* Schaeffer
*Oxygonus obesus* Say**
*Paractenicera fulvipes* (Bland)
*Prosternon medianum* (Germar)
*Pseudanostirus hamatus* (Say)
*Pseudanostirus hieroglyphicus* (Say)
*Pseudanostirus nigricollis *(Bland)
*Pseudanostirus propolus* (LeConte)
*Pseudanostirus triundulatus* (Randall)
*Selatosomus appropinquans* (Randall)
*Selatosomus pulcher* (LeConte)
*Selatosomus splendens* (Ziegler)
*Setasomus atratus* (LeConte)
*Setasomus nitidulus* (LeConte)
*Setasomus rufopleuralis* (Fall)
*Sylvanelater cylindriformis* (Herbst)
**Tribe Hypnoidini Schwarz**
*Hypnoidus abbreviatus* (Say)
*Hypnoidus bicolor* (Eschscholtz)
*Ligmargus lecontei* (Leng)*
*Margaiostus grandicollis* (LeConte)
**Subfamily Negastriinae Nakane & Kishii**
*Microhypnus striatulus* (LeConte)
*Negastrius arnetti* Stibick
*Negastrius delumbis* (Horn)
*Negastrius atrosus* Wells**
*Neohypdonus tumescens* (LeConte)
*Oedostethus femoralis* LeConte
*Paradonus olivereae* Stibick
*Paradonus pectoralis* (Say)*
*Zorochrus melsheimeri* (Horn)
**Subfamily Elaterinae Leach**
**Tribe Agriotini Laporte**
*Agriotes collaris *(LeConte)
*Agriotes fuscosus* (LeConte)
*Agriotes limosus* (LeConte)
*Agriotes mancus* (Say)
*Agriotes quebecensis* Brown*
*Agriotes sputator* (Linnaeus)
*Agriotes pubescens* Melsheimer*
*Agriotes stabilis* (LeConte)
*Dalopius cognatus* Brown
*Dalopius fuscipes* Brown
*Dalopius pallidus* Brown
*Dalopius vagus* Brown
*Dalopius brevicornis* Brown**
**Tribe Ampedini Gistel**
*Ampedus apicatus* (Say)
*Ampedus areolatus* (Say)*
*Ampedus collaris* (Say)
*Ampedus deletus* (LeConte)
*Ampedus evansi* Brown
*Ampedus fusculus* (LeConte)
*Ampedus laurentinus* Brown
*Ampedus luctuosus* (LeConte)
*Ampedus minipennis* (LeConte)
*Ampedus mixtus* (Herbst)
*Ampedus molestus* (LeConte)
*Ampedus nigricans* (Germar)
*Ampedus nigricollis* (Herbst)*
*Ampedus nigrinus* (Herbst)
*Ampedus oblessus* (Say)
*Ampedus protervus* (LeConte)*
*Ampedus pullus *Germar
*Ampedus rubricus* (Say)
*Ampedus sanguinipennis* (Say)
*Ampedus sayi* (LeConte)
*Ampedus semicinctus* (Randall)
*Ampedus subtilis* (LeConte)
*Ampedus vitiosus* (LeConte)
**Tribe Elaterini Leach**
*Elater abruptus* Say*
*Sericus honestus* (Randall)
*Sericus incongruus* (LeConte)
*Sericus viridanus* (Say)*
**Tribe Megapenthini Gurjeva**
*Megapenthes rogersi* Horn
*Megapenthes stigmosus* (LeConte)
*Megapenthes solitarius* Fall**
**Tribe Melontini Candèze**
*Melanotus castanipes* (Paykull)
*Melanotus decumanus *(Erichson)
*Melanotus leonardi *(LeConte)**
*Melanotus similis *(Kirby)
*Melanotus sagittarus* (LeConte)**
**Tribe Pomachiliini Candèze**
*Agriotella bigeminata* (Randall)
*Agriotella debilis* (LeConte)
**Subfamily Cardiophorinae Candèze**
*Cardiophorus convexulus* LeConte
*Cardiophorus gagates *Erichson
*Cardiophorus propinquus *Lanchester

Notes: *New to province, **New to Maritime provinces.

### Species accounts

All records below are species newly recorded for New Brunswick, Canada unless noted otherwise (additional records). Species followed by ** are newly recorded from the Maritime provinces of Canada.

The classification of the Elateridae follows Bouchard et al. (2011).

### Family Elateridae Leach, 1815

Subfamily Agrypninae Candèze, 1857

Tribe Agrypnini Candèze, 1857

#### Danosoma obtectum

(Say, 1839)**

http://species-id.net/wiki/Danosoma_obtectum

Map 1

##### Material examined.

**New Brunswick, York Co.**, 14 km WSW of Tracy, S of Rt. 645, 45.6741°N, 66.8661°W, 13–27.VII.2010, R. Webster & C. MacKay, old mixed forest with red and white spruce, red and white pine, balsam fir, eastern white cedar, red maple, and *Populus* sp., Lindgren funnel trap (1, RWC).

##### Collection and habitat data.

The single specimen from New Brunswick was captured during July in a Lindgren funnel trap deployed in an old mixed forest.

##### Distribution in Canada and Alaska.

YK, NT, BC, AB, SK, MB, ON, PQ, **NB** (Bousquet 1991). Majka and Johnson (2008) removed *Danosoma obtectum* from the faunal list of Nova Scotia due to a lack of a supporting voucher specimen.

**Map 1.**
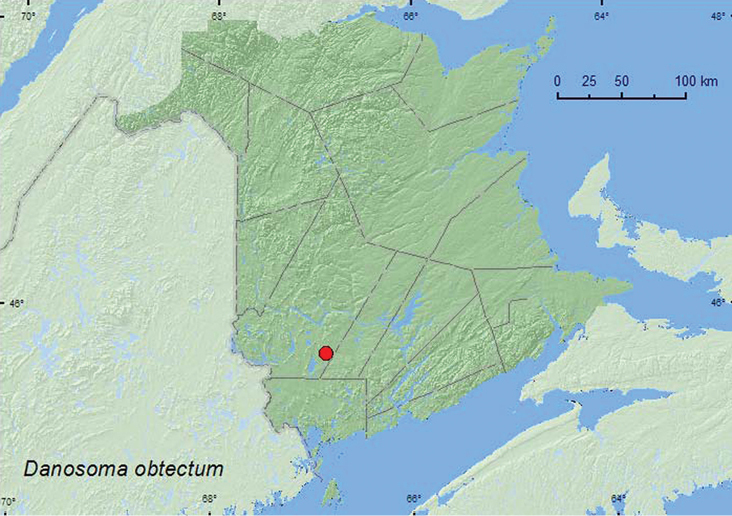
Collection localities in New Brunswick, Canada of *Danosoma obtectum.*

### Subfamily Dendrometrinae Gistel, 1848

Tribe Dendrometrini Gistel, 1848

#### Athous posticus

(Melsheimer, 1846)

http://species-id.net/wiki/Athous_posticus

Map 2

##### Material examined.

**New Brunswick, Carleton Co.**, Meduxnekeag Valley Nature Preserve, 46.1957°N, 67.6803°W, 22.VII.2004, J. Edsall & R. P. Webster, mixed forest, u.v. light (1, RWC); Jackson Falls, Bell Forest, 46.2200°N, 67.7231°W, 5–12.VII.2008, 19–28.VII.2008, R. P. Webster, mature hardwood forest, Lindgren funnel traps (5, AFC, RWC). **Queens Co.**, Cranberry Lake P.N.A. (Protected Natural Area), 46.1125°N, 65.6075°W, 1–10.VII.2009, 15–21.VII.2009, 21–28.VII.2009, R. Webster & M.-A. Giguère, old red oak forest, Lindgren funnel traps (5, AFC); same locality data and forest type, 20.VII-4.VIII.2011, M. Roy & V. Webster, Lindgren funnel traps in forest canopy (3, NBM, RWC). **York Co.**, 15 km W of Tracy off Rt. 645, 45.6848°N, 66.8821°W, 14–20.VII.2009, R. Webster & M.-A. Giguère, old red pine forest, Lindgren funnel trap (1, RWC); same locality and habitat data but 30.VI–13.VII.2010, R. Webster & K. Burgess, Lindgren funnel trap (1, RWC); 14 km WSW of Tracy, S of Rt. 645, 45.6741°N, 66.8661°W, 16–30.VI.2010, 30.VI–13.VII.2010, R. Webster, C. MacKay, & K. Burgess, old mixed forest with red and white spruce, red and white pine, balsam fir, eastern white cedar, red maple, and *Populus* sp., Lindgren funnel traps (3, AFC, RWC).

##### Collection and habitat data.

One adult was collected at an ultraviolet light, but most individuals were captured in Lindgren funnel traps in mixed and old mixed forests, a mature hardwood forest, an old red oak (*Quercus rubra* L.) forest, and an old red pine (*Pinus resinosa* Ait.) forest. Adults were collected during June, July, and August.

##### Distribution in Canada and Alaska.

ON, QC, **NB**, NS (Bousquet 1991; Majka and Johnson 2008).

**Map 2.**
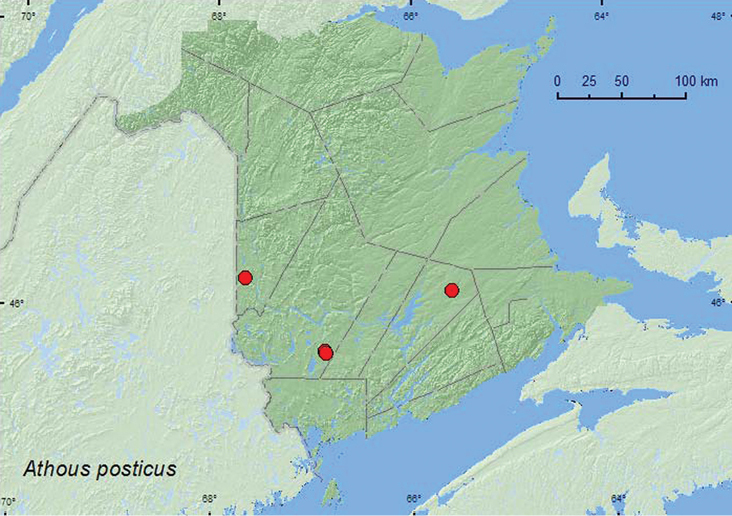
Collection localities in New Brunswick, Canada of *Athous posticus.*

#### Athous scapularis

(Say, 1839)

http://species-id.net/wiki/Athous_scapularis

Map 3

##### Material examined. 

**New Brunswick, Carleton Co.**, Jackson Falls, Bell Forest, 46.2208°N, 67.7211°W, 28.VI.2005, R. P. Webster, mature hardwood forest, u.v. light (1, RWC); same locality but 46.2200°N, 67.7231°W, 5–12.VII.2008, R. P. Webster, mature hardwood forest, Lindgren funnel trap (1, RWC). **Queens Co.**, Cranberry Lake P.N.A., 46.1125°N, 65.6075°W, 18–31.VIII.2011, M. Roy & V. Webster, old red oak forest, Lindgren funnel trap (1, AFC).

##### Collection and habitat data.

Adults of this species were found in a mature hardwood forest with American beech (*Fagus grandifolia* Ehrh.), sugar maple (*Acer saccharum* Marsh.), and white ash (*Fraxinus americana* L.) and in an old red oak forest. Adults were captured at an ultraviolet light and in Lindgren funnel traps. This species was captured during June, July, and August. Becker (1974) reported larvae of this species from forest litter and decaying logs.

##### Distribution in Canada and Alaska.

ON, QC, **NB**, NS (Bousquet 1991; Majka and Johnson 2008).

**Map 3.**
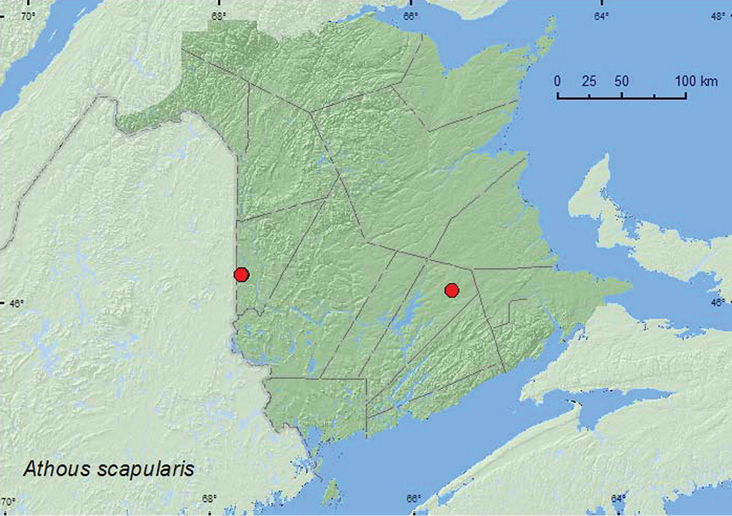
Collection localities in New Brunswick, Canada of *Athous scapularis.*

#### Elathous discalceatus

(Say, 1839)

http://species-id.net/wiki/Elathous_discalceatus

Map 4

##### Material examined.

**New Brunswick, Carleton Co.**, Jackson Falls, Bell Forest, 46.2200°N, 67.7231°W, 19–28.VII.2008, R. P. Webster, mature hardwood forest, Lindgren funnel trap (1, RWC). **York Co.**, 15 km W of Tracy off Rt. 645, 45.6848°N, 66.8821°W, 4–11.VIII.2009, R. Webster & M.-A. Giguère, old red pine forest, Lindgren funnel trap (1, RWC); same locality data, 27.VII–10.VIII.2010, R. Webster & C. Hughes, Lindgren funnel traps (3, AFC, RWC).

##### Collection and habitat data.

Adults were captured during late July and August in Lindgren funnel traps in a mature hardwood forest with American beech, sugar maple, and white ash, and in an old red pine forest.

##### Distribution in Canada and Alaska.

ON, QC, **NB**, NS (Bousquet 1991).

**Map 4.**
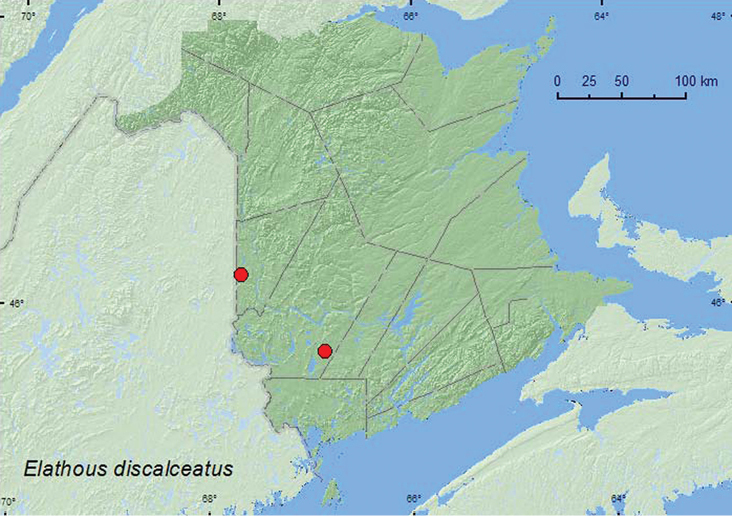
Collection localities in New Brunswick, Canada of *Elathous discalceatus.*

#### Hemicrepidius memnonius

(Herbst, 1806)

http://species-id.net/wiki/Hemicrepidius_memnonius

Map 5

##### Material examined.

**New Brunswick, York Co.**, Fredericton, 27.VII.1929, R. P. Gorham, (2, AFC); Charters Settlement, 45.8395°N, 66.7391°W, 1.VIII.2007, R. P. Webster, mixed forest, u.v. light (1, RWC); 15 km W of Tracy off Rt. 645, 45.6848°N, 66.8821°W, 20–29.VII.2009, 29.VII-4.VIII.2009, 11–18.VIII.2009, R. Webster & M.-A. Giguère, old red pine forest, Lindgren funnel traps (8, AFC, RWC); same locality data but 13–27.VII.2010, R. Webster & C. MacKay, Lindgren funnel traps (2, AFC, RWC).

##### Collection and habitat data.

*Hemicrepidius memnonius* was collected at an ultraviolet light in a mixed forest and from Lindgren funnel traps in an old red pine forest. Adults were captured during July and August.

##### Distribution in Canada and Alaska.

AB, SK, MB, ON, QC, **NB**, NS, PE (Bousquet 1991).

**Map 5.**
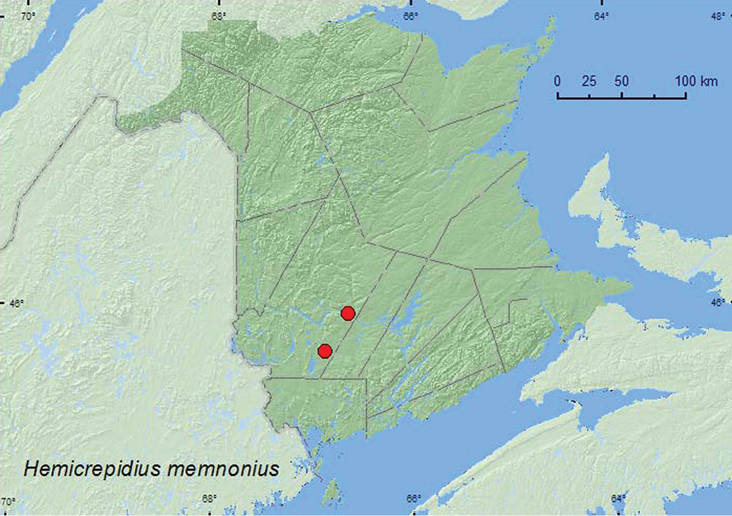
Collection localities in New Brunswick, Canada of *Hemicrepidius memnonius.*

### Tribe Prosternini Gistel, 1856

#### Hypoganus sulcicollis

(Say, 1834)

http://species-id.net/wiki/Hypoganus_sulcicollis

Map 6

##### Material examined.

**New Brunswick, Carleton Co.**, Jackson Falls, Bell Forest, 46.2199°N, 67.7231°W, 9.IX.2006, 6.V.2007, R. P. Webster, mature hardwood forest, under bark of fallen beech logs (2, RWC); same locality but 20–26.V.2009, R. Webster & M.-A. Giguère, mature hardwood forest, Lindgren funnel trap (1, RWC). **Queens Co.**, Grand Lake near Scotchtown, 45.8762°N, 66.1816°W, 25.IV.2004, R. Webster & M.-A. Giguère, oak and maple forest, under bark of oak (1, RWC); Cranberry Lake P.N.A., 46.1125°N, 65.6075°W, 21–27.V.2009, 10–15.VII.2009, R. Webster & M.-A. Giguère, old red oak forest, Lindgren funnel traps (2, RWC); Grand Lake Meadows P.N.A., 45.8227°N, 66.1209°W, 3–21.VI.2011, 21.VI–5.VII.2011, 5–19.VII.2011, 5–17.VIII,2011, M. Roy & V. Webster, old silver maple forest with green ash and seasonally flooded marsh, Lindgren funnel traps in forest canopy (4, NBM, RWC). **Sunbury Co.**, Burton near Sunpoke Lake, 45.7663°N, 66.5550°W, 20.VII.2006, R. P. Webster, oak forest, under loose bark of red oak (1, RWC).

##### Collection and habitat data.

*Hypoganus sulcicollis* (Say) was collected in a mature hardwood forest with American beech, sugar maple, and white ash, in a red oak and red maple (*Acer rubrum* L.) forest, an old silver maple (*Acer saccharinum* L.) forest, and in an old red oak forest. Adults were collected from under bark of fallen American beech, under bark of red oak, and from Lindgren funnel traps. Adults were captured during April, May, June, July, August, and September.

##### Distribution in Canada and Alaska.

MB, ON, QC, **NB**, NS (Bousquet 1991; Majka and Johnson 2008).

**Map 6.**
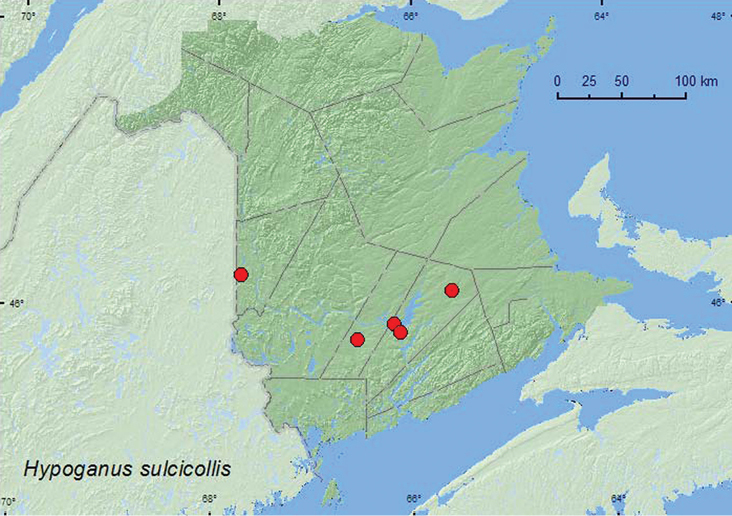
Collection localities in New Brunswick, Canada of *Hypoganus sulcicollis.*

#### Hypoganus rotundicollis

(Say, 1825)**

http://species-id.net/wiki/Hypoganus_rotundicollis

Map 7

##### Material examined.

**New Brunswick, Queens Co.**, near “Trout Creek”, 45.8237°N, 66.1225°W, 6.IX.2007, R. P. Webster, silver maple swamp, sweeping foliage on margin of marsh (1, RWC).

##### Collection and habitat data.

The sole New Brunswick specimen of this species was collected during September by sweeping marsh vegetation on the margin of a silver maple swamp.

##### Distribution in Canada and Alaska.

ON, **NB** (Bousquet 1991).

**Map 7.**
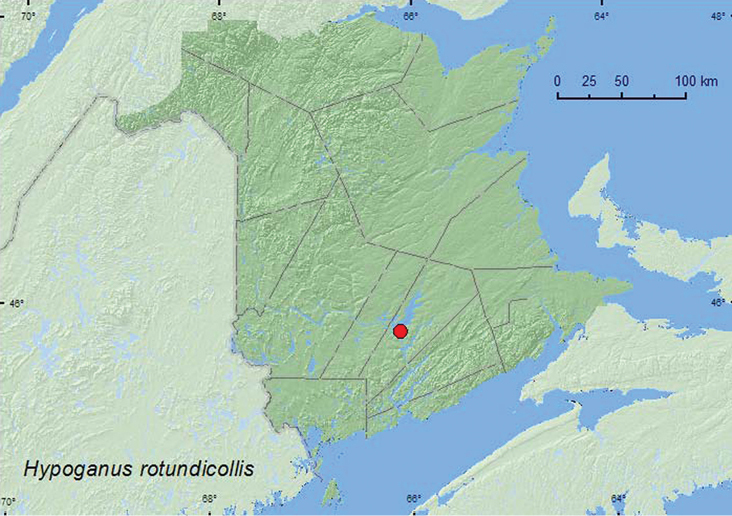
Collection localities in New Brunswick, Canada of *Hypoganus rotundicollis.*

#### Oxygonus obesus

(Say, 1823)**

http://species-id.net/wiki/Oxygonus_obesus

Map 8

##### Material examined.

**New Brunswick, York Co.**, Canterbury, Browns Mountain Fen, 45.8967°N, 67.6343°W, 1.VI.2005, R. Webster & M.-A. Giguère, calcareous fen with shrubby cinquefoil, sweeping (2, RWC).

##### Collection and habitat data.

Specimens of this species were swept from vegetation in an open calcareous cedar fen with shrubby cinquefoil (*Pentaphylloides floribunda* (Pursh) A. Löve) during early June.

##### Distribution in Canada and Alaska.

AB, MB, ON, QC, **NB** (Bousquet 1991).

**Map 8.**
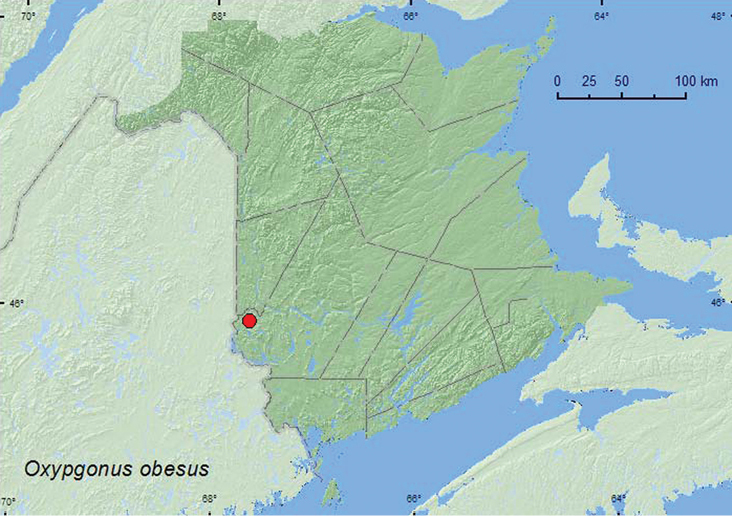
Collection localities in New Brunswick, Canada of *Oxygonus obesus.*

### Tribe Hypnoidini Schwarz, 1906 (1860)

#### Ligmargus lecontei

(Leng, 1918)

http://species-id.net/wiki/Ligmargus_lecontei

Map 9

##### Material examined.

**New Brunswick, Restigouche Co.**, Jacquet River Gorge P.N.A. near Jacquet R., 47.8897°N, 66.0835°W, 23.VI.2008, 26.VI.2008, R. P. Webster, river margin, among cobblestones (2, RWC); same locality but 47.8204°N, 66.0833°W, 14.VI.2009, R. P. Webster, river margin, among cobblestones (1, RWC); same locality but 47.8357°N, 66.0779°W, 14.V.2010, 24.V.2010, R. P. Webster, partially shaded gravel bar near confluence of brook and river, among cobblestones (2, RWC).

##### Collection and habitat data.

*Ligmargus lecontei* adults were collected from under cobblestones along the margin of a fast-flowing, clear (cool water), rocky, river during May and June.

##### Distribution in Canada and Alaska.

ON, QC, **NB**, NS (Bousquet 1991).

**Map 9.**
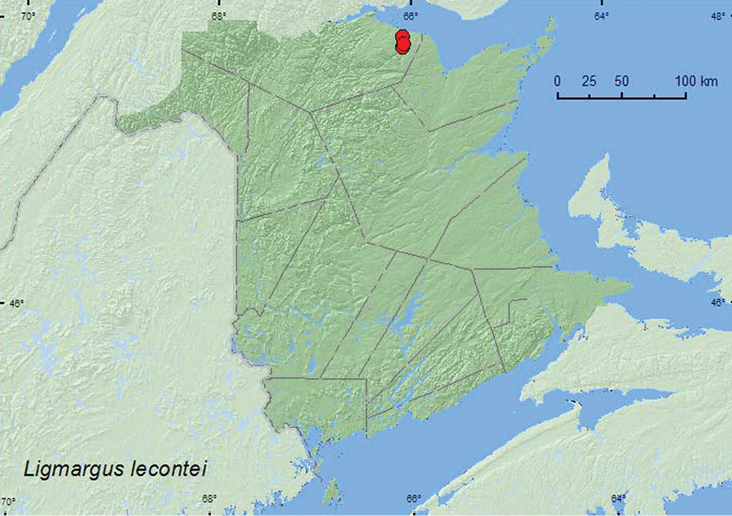
Collection localities in New Brunswick, Canada of *Ligmargus lecontei.*

### Subfamily Negastriinae Nakane and Kishii, 1956

***Negastrius exiguus* (Randall, 1838)**

The record of *Negastrius exiguus* in Majka and Johnson (2008) was based on a misidentification by C.G. Majka and was *Negastrius atrosus* Wells (determined by Serge Laplante). In view of this, *Negastrius exiguus* is removed from the faunal list of New Brunswick.

#### Negastrius atrosus

Wells, 1996**

http://species-id.net/wiki/Negastrius_atrosus

Map 10

##### Material examined.

**New Brunswick, Queens Co.**, Bayard, at Nerepis River, 45.4426°N, 66.3280°W, 30.V.2008, R. P. Webster, river margin, under small rocks embedded in gravel (2, RWC). **Restigouche Co.**, confluence of Restigouche River and Stillwater Brook, 26.VI.2000, R. Webster, F. Roy, & P. Poitras, in gravel on river margin (1, RWC). **York Co.**, Rt. 105 at Nashwaaksis River, 45.9853°N, 66.6910°W, 9.V.2006, R. P. Webster, river margin, splashing water onto sand bar (2, RWC); 1.5 km S of Taymouth at the Nashwaak River, 46.1582°N, 66.6134°W, 15.VI.2008, R. P. Webster, on sand bar under drift material on sand (5, RWC).

##### Collection and habitat data.

*Negastrius atrosus* adults were collected along river margins from under small rocks, in gravel, and under drift material on sand bars. Two adults were collected by splashing water onto sand on a sand bar. Adults were collected during May and June. No details on the habitat requirements of this species were given in Wells (1996). Wells (1996) reported that other species of *Negastrius* were associated with riparian habitats and inhabit sandy–to–rocky stream and river margins.

##### Distribution in Canada and Alaska.

ON, QC, **NB** (Wells 1996).

**Map 10.**
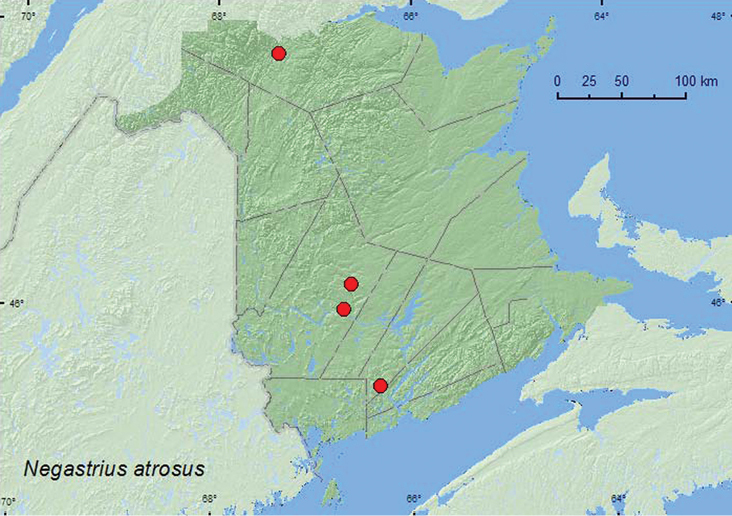
Collection localities in New Brunswick, Canada of *Negastrius atrosus.*

#### Paradonus pectoralis

(Say, 1839)

http://species-id.net/wiki/Paradonus_pectoralis

Map 11

##### Material examined.

**New Brunswick, Charlotte Co.**, St. Andrews, 45.0751°N, 67.0374°W, 25.VIII.2006, R. P. Webster, sea beach, sweeping foliage (1, RWC). **Queens Co.**, Grand Lake near Scotchtown, 45.8762°N, 66.1816°W, 5.VI.2004, R. P. Webster, in gravel near shoreline of lake (2, RWC); Grand Lake at Stony Point, 46.0031°N, 66.0337°W, 17.VIII.2004, D. Sabine & R. Webster, lakeshore, cobblestone beach, among cobblestones (3, RWC). **York Co.**, Charters Settlement, 45.8395°N, 66.7391°W, 19.VII.2005, 9.VII.2006, 17.VII.2008, R. P. Webster, mixed forest, u.v. light (3, RWC).

##### Collection and habitat data.

Adults of this species were collected in gravel and among cobblestones along lakeshores, by sweeping foliage on a sea beach, and at an ultraviolet light in a mixed forest. Adults were collected during June, July, and August.

##### Distribution in Canada and Alaska.

BC, AB, SK, MB, ON, QC, **NB**, NS (Bousquet 1991).

**Map 11.**
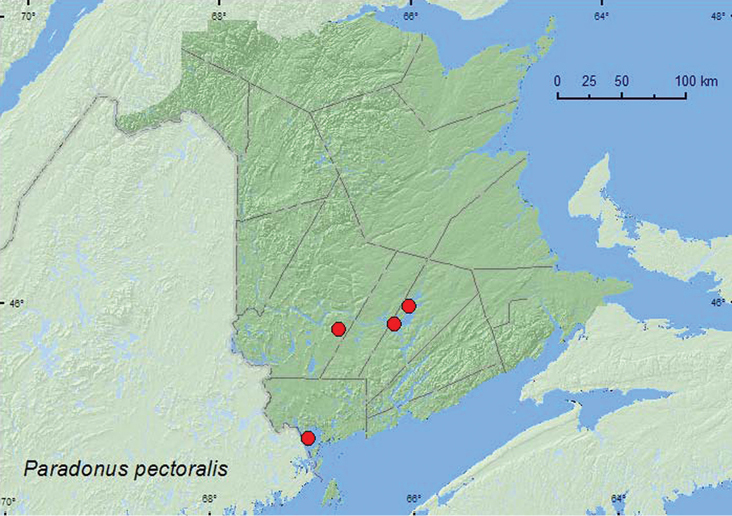
Collection localities in New Brunswick, Canada of *Paradonus pectoralis.*

### Subfamily Elaterinae Leach, 1815

Tribe Agriotini Laporte, 1840

#### Agriotes quebecensis

Brown, 1933

http://species-id.net/wiki/Agriotes_quebecensis

Map 12

##### Material examined.

**New Brunswick, Restigouche Co.**, Jacquet River Gorge P.N.A., 47.7235°N, 66.1278°W, 16.VI.2009, K. A. A. Vandenbroeck (1, NBM). **York Co.**, Charters Settlement, 45.8380°N, 66.7310°W, 14.V.2004, R. P. Webster, beating foliage (1, RWC); 14 km WSW of Tracy, S of Rt. 645, 45.6741°N, 66.8661°W, 17–31.V.2010, R. Webster & C. MacKay, old mixed forest with red and white spruce, red and white pine, balsam fir, eastern white cedar, red maple, and *Populus* sp., Lindgren funnel trap (1, AFC).

##### Collection and habitat data.

One individual was beaten from foliage in a mixed forest, another was captured in a Lindgren funnel trap deployed in an old mixed forest. Adults were captured during May and June.

##### Distribution in Canada and Alaska.

MB, ON, QC, **NB**, PE, NS (Bousquet 1991; Majka and Johnson 2008).

**Map 12.**
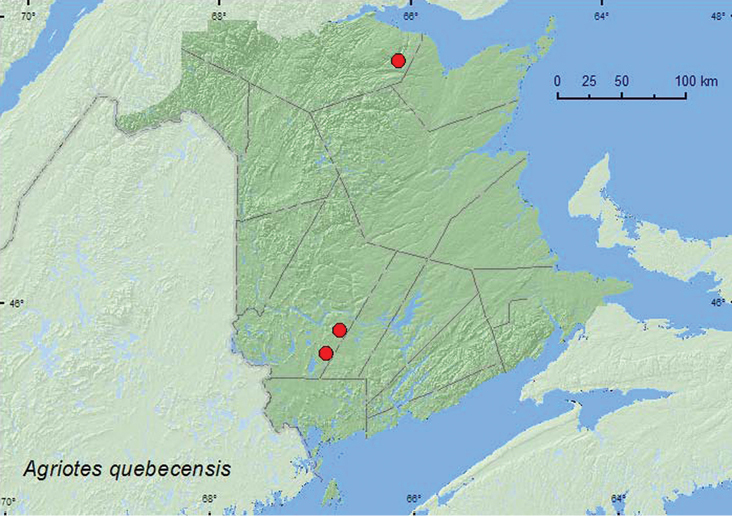
Collection localities in New Brunswick, Canada of *Agriotes quebecensis.*

#### Agriotes pubescens

Melsheimer, 1845

http://species-id.net/wiki/Agriotes_pubescens

Map 13

##### Material examined.

**New Brunswick, Queens Co.**, Grand Lake Meadows P.N.A., 45.8227°N, 66.1209°W, 31.V–15.VI.2010, 15–29.VI.2010, R. Webster & C. MacKay, old silver maple forest with green ash and seasonally flooded marsh, Lindgren funnel traps (5, AFC, RWC). **Sunbury Co.**, Burton near Sunpoke Lake, 45.7658°N, 66.5546°W, 20.VI.2007, R. P. Webster, red oak and red maple forest, on foliage of *Quercus rubra* (1, RWC).

##### Collection and habitat data.

Adults were collected during June from Lindgren funnel traps in an old silver maple forest (swamp) and from foliage of red oak in a red oak and red maple stand. Both forest sites were near seasonally flooded marshes. 

##### Distribution in Canada and Alaska.

MB, ON, QC, **NB** (Bousquet 1991). Bousquet (1991) reported *Agriotes pubescens* Melsheimer from New Brunswick. Majka and Johnson (2008) were unable to locate voucher specimens to support the record and, thus, they removed it from the faunal list of New Brunswick. The records above establish the presence of this species in the province.

**Map 13.**
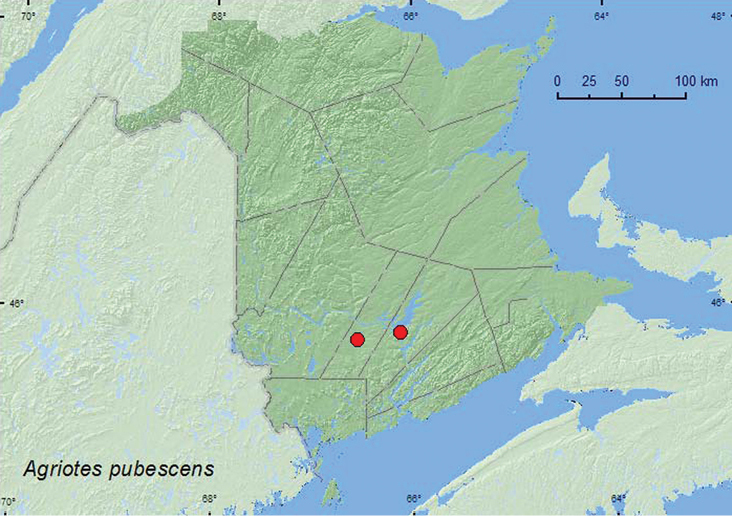
Collection localities in New Brunswick, Canada of *Agriotes pubescens.*

#### Dalopius brevicornis

Brown, 1934**

http://species-id.net/wiki/Dalopius_brevicornis

Map 14

##### Material examined.

**New Brunswick, Carleton Co.**, Jackson Falls, Bell Forest, 46.2152°N, 67.7190°W, 1.VI.2005, M.-A. Giguère & R. P. Webster, upper river margin near floodplain forest, sweeping foliage (4, RWC); Meduxnekeag Valley Nature Preserve, 46.1931°N, 67.6825°W, 8. VI.2005, M.-A. Giguère & R. P. Webster, margin of floodplain forest with butternut, sweeping (3, RWC).

##### Collection and habitat data.

Adults were collected during early June by sweeping foliage near floodplain forests adjacent to rivers.

##### Distribution in Canada and Alaska.

ON, QC, **NB** (Bousquet 1991).

**Map 14.**
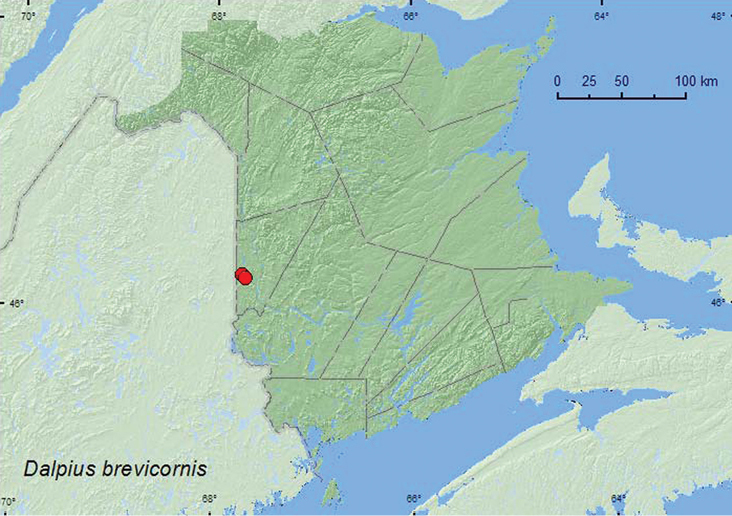
Collection localities in New Brunswick, Canada of *Dalopius brevicornis.*

### Tribe Ampedini Gistel, 1848

#### Ampedus areolatus

(Say, 1823)

http://species-id.net/wiki/Ampedus_areolatus

Map 15

##### Material examined.

**New Brunswick, Carleton Co.**, Jackson Falls, Bell Forest, 46.2152°N, 67.7190°W, 12.VI.2008, R. P. Webster, river margin, treading vegetation in seepage area (1, RWC); Meduxnekeag Valley Nature Preserve, 46.1931°N, 67.6825°W, 8.VI.2005, M.-A. Giguère & R. P. Webster, floodplain forest with butternut, sweeping (1, RWC). **Queens Co.**, Grand Lake Meadows P.N.A., 45.8227°N, 66.1209°W, 19–31.V.2010, 31.V–15.VI.2010, 15–29.VI.2010, 29.VI–12.VII.2010, R. Webster, C. MacKay, M. Laity, & R. Johns, old silver maple forest with green ash and seasonally flooded marsh, Lindgren funnel traps (18, AFC, RWC); same locality data and forest type, 17–30.VIII.2011, C. Hughes & R. P. Webster, Lindgren funnel traps (2, NBM).

##### Collection and habitat data.

Adults were collected by treading vegetation in a seepage area along a river margin, by sweeping vegetation in a floodplain forest, and from Lindgren funnel traps deployed in an old silver maple forest near a seasonally flooded marsh. Adults were collected during May, June, July, and August.

##### Distribution in Canada and Alaska.

ON, QC, **NB**, NS (Bousquet 1991; Majka and Johnson 2008).

**Map 15.**
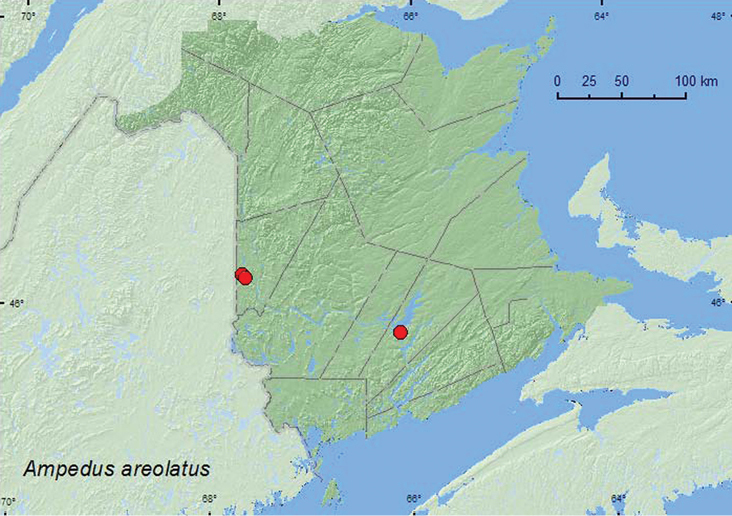
Collection localities in New Brunswick, Canada of *Ampedus areolatus.*

#### Ampedus nigricollis

(Herbst, 1801)

http://species-id.net/wiki/Ampedus_nigricollis

Map 16

##### Material examined.

**New Brunswick, Queens Co.,** Grand Lake Meadows P.N.A., 45.8227°N, 66.1209°W, 21.VI-5.VII.2011, M. Roy & V. Webster, old silver maple forest with green ash and seasonally flooded marsh, Lindgren funnel traps in forest canopy (1, RWC). **Sunbury Co.**, Maugerville, Portobello Creek N.W.A., 45.8990°N, 66.4200°W, 28.VI.2004, R. P. Webster, silver maple swamp, under bark of silver maple (1, RWC).

##### Collection and habitat data.

One specimen was found under bark of a silver maple during late June in a silver maple swamp, another was captured between late June and early July in a Lindgren funnel trap deployed in the canopy of a silver maple in a silver maple swamp. Majka and Johnson (2008) reported this species from rotten wood of poplar, a spruce stump, and reared from an apple log in Nova Scotia.

##### Distribution in Canada and Alaska.

ON, QC, **NB**, NS (Bousquet 1991; Majka and Johnson 2008).

**Map 16.**
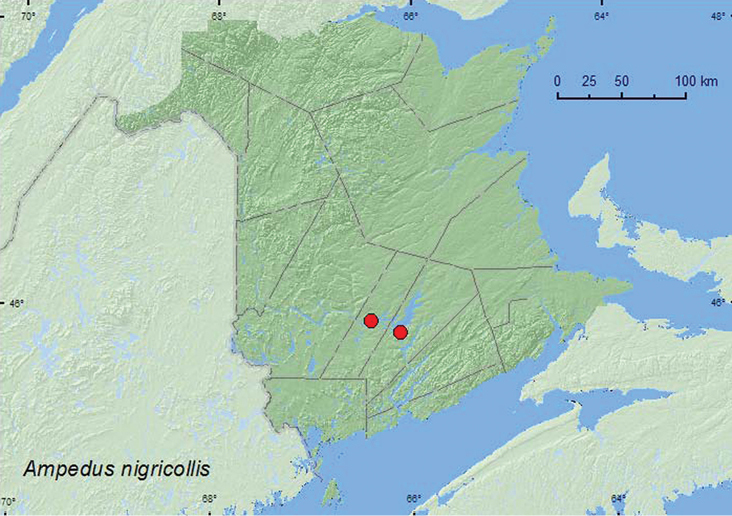
Collection localities in New Brunswick, Canada of *Ampedus nigricollis.*

#### Ampedus oblessus

(Say, 1833)

http://species-id.net/wikiAmpedus_oblessus

Map 17

##### Material examined.

**Additional New Brunswick records, Queens Co.**, Cranberry Lake P.N.A, 46.1125°N, 65.6075°W, 5–11.VI.2009, 18–25.VI.2009, R. Webster & M.-A. Giguère, old red oak forest, Lindgren funnel traps (3, AFC, RWC); same locality data and forest type, 13–25.V.2011, 25.V–7.VI.2011, 7–22.VI.2011, 29.VI–7.VII.2011, M. Roy & V. Webster, Lindgren traps in forest canopy (68, AFC, CNC, NBM, RWC); Grand Lake Meadows P.N.A., 45.8227°N, 66.1209°W, 1–3.VI.2011, 3–21.VI.2011, 21.VI–5.VII.2011, 5–19.VII.2011, M. Roy & V. Webster, old silver maple forest with green ash and seasonally flooded marsh, Lindgren funnel traps in forest canopy (13, AFC, NBM, RWC).

##### Collection and habitat data.

Adults were captured during May, June, and July in Lindgren funnel traps in an old red oak stand and an old silver maple swamp. Most (77 out of 81) individuals were captured in traps deployed in the forest canopy (mid crown).

##### Distribution in Canada and Alaska.

AB, SK, MB, ON, QC, NB (Bousquet 1991). *Ampedus oblessus* (Say) was reported for New Brunswick in Bousquet (1991) but was not listed as a member of the fauna by Majka and Johnson (2008). The above record confirms the presence of this species for the province.

**Map 17.**
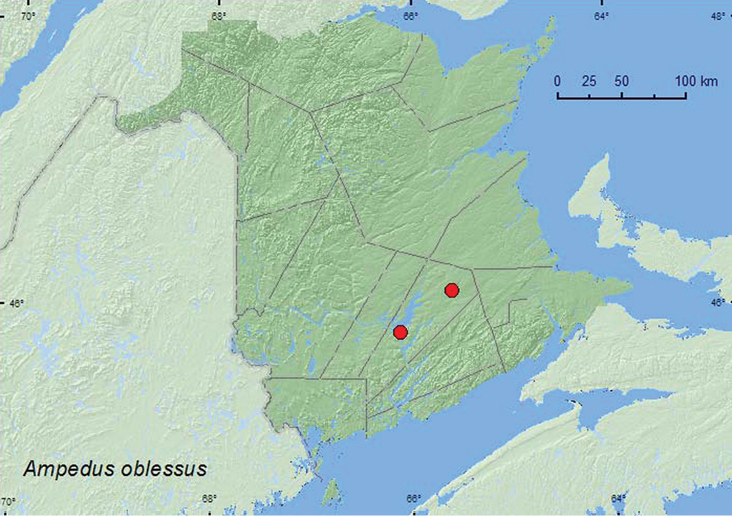
Collection localities in New Brunswick, Canada of *Ampedus oblessus.*

#### Ampedus protervus

(LeConte, 1853)

http://species-id.net/wiki/Ampedus_protervus

Map 18

##### Material examined.

**New Brunswick, Carleton Co.**, Jackson Falls, Bell Forest, 46.2200°N, 67.7231°W, 9.IX.2006, 6.V.2007, R. P. Webster, mature hardwood forest, under bark of fallen beech log (3, RWC); same locality but 4–12.VI.2008, 12–19.VI.2008, 19–27.VI.2008, R. P. Webster, mature hardwood forest, Lindgren funnel traps (3, AFC, RWC); same locality and habitat data, 14–20.V.2009, M.-A. Giguère & R. Webster, Lindgren funnel traps (2, RWC). **Queens Co.**, Cranberry Lake P.N.A, 46.1125°N, 65.6075°W, 12–21.V.2009, 1–10.VII.2009, R. Webster & M.-A. Giguère, old red oak forest, Lindgren funnel traps (2, AFC, RWC); same locality data and forest type, 25.V-7.VI.2011, 7–22.VI.2011, 29.VI–7.VII.2011, M. Roy & V. Webster, Lindgren funnel traps (3, AFC, NBM). **York Co.**,Charters Settlement, 45.8331°N, 66.7410°W, 27.VII.2005, R. P. Webster, mixed forest, on foliage of *Alnus incana* (1, RWC); 15 km W of Tracy off Rt. 645, 45.6848°N, 66.8821°W, 14–20.VII.2009, R. Webster & M.-A. Giguère, old red pine forest, Lindgren funnel trap (1, RWC).

##### Collection and habitat data.

Adults of this species were captured in Lindgren funnel traps deployed in mature hardwood forests with American beech, sugar maple, and white ash, an old red oak forest, and an old red pine forest. Adults were also collected from under bark of a fallen beech log in mature hardwood forest and from alder (*Alnus incana* (L.) Moench) foliage in a mixed forest. Adults were collected during May, June, July, and September.

##### Distribution in Canada and Alaska.

ON, QC, **NB**, NS (Bousquet 1991).

**Map 18.**
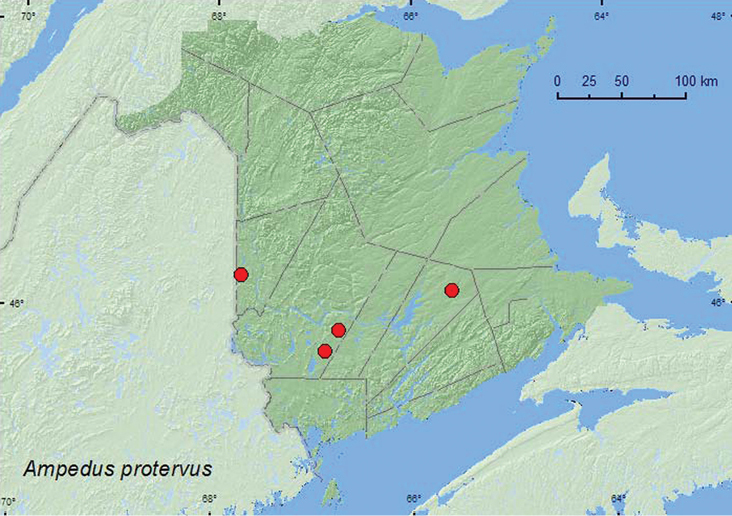
Collection localities in New Brunswick, Canada of *Ampedus protervus.*

### Tribe Elaterini Leach, 1915

#### Elater abruptus

Say, 1825

http://species-id.net/wiki/Elater_abruptus

Map 19

##### Material examined.

**New Brunswick, Carleton Co.**, Jackson Falls, Bell Forest, 46.2200°N, 67.7231°W, 19–28.VII.2008, R. P. Webster, mature hardwood forest, Lindgren funnel trap (1, RWC). **Queens Co.**, Cranberry Lake P.N.A., 46.1125°N, 65.6075°W, 28.VII–6.VIII.2009, R. Webster & M.-A. Giguère, old red oak forest, Lindgren funnel trap (1, AFC); same locality data and forest type, 20.VII-4.VIII.2011, 4–18.VIII.2011, M. Roy & V. Webster, Lindgren funnel traps in forest canopy (5, AFC, NBM, RWC); Grand Lake Meadows P.N.A., 45.8227°N, 66.1209°W, 19.VII-5.VIII.2011, 5–17.VII.2011, M. Roy & V. Webster, old silver maple forest and seasonally flooded marsh, Lindgren funnel traps in forest canopy (8, AFC, NBM, RWC).

##### Collection and habitat data.

Adults were captured in Lindgren funnel traps in a mature hardwood forest with American beech, sugar maple, and white ash, in an old silver maple forest, and in an old red oak forest. Most adults were captured in traps deployed in the forest canopy. Adults were captured during July and August.

##### Distribution in Canada and Alaska.

ON, QC, **NB**, NS (Bousquet 1991).

**Map 19.**
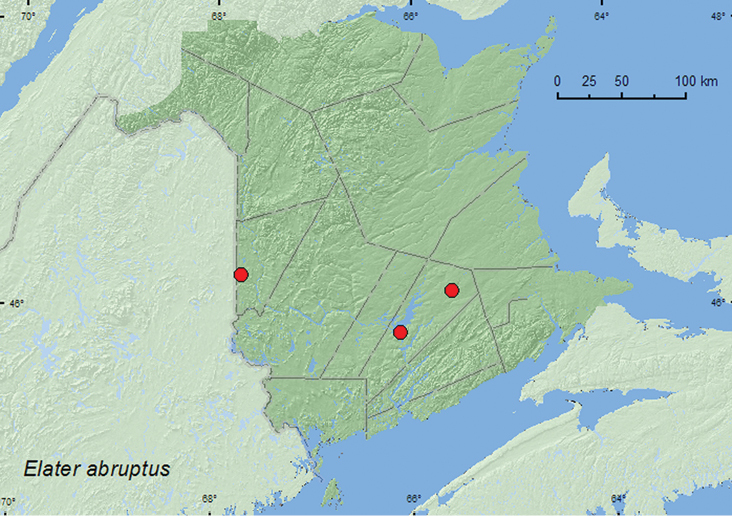
Collection localities in New Brunswick, Canada of *Elater abruptus.*

#### Sericus viridanus

(Say, 1825)

http://species-id.net/wiki/Sericus_viridanus

Map 20

##### Material examined.

**New Brunswick, Carleton Co.**, Jackson Falls, Bell Forest, 46.2200°N, 67.7231°W, 12–29.VI.2008, R. P. Webster, mature hardwood forest, Lindgren funnel trap (1, RWC).

##### Collection and habitat data.

The only specimen known from New Brunswick was captured during June in a Lindgren funnel trap deployed in a mature hardwood forest with American beech, sugar maple, and white ash.

##### Distribution in Canada and Alaska.

ON, QC, **NB**, NS (Bousquet 1991).

**Map 20.**
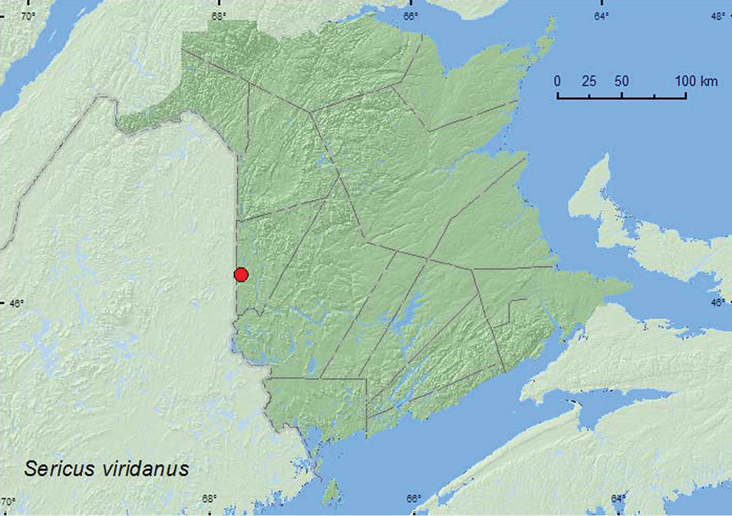
Collection localities in New Brunswick, Canada of *Sericus viridanus.*

### Tribe Megapenthini Gurjeva, 1973

#### Megapenthes rogersi

Horn, 1871

http://species-id.net/wiki/Megapenthes_rogersi

Map 21

##### Material examined.

**Additional New Brunswick records, Kings Co.**, Hampton, Hampton Marsh, 45.4787°N, 65.9007°W, 13.VII.2005, R. P. Webster, floodplain forest, on foliage of silver maple (1, RWC). **Queens Co.**, Cranberry Lake P.N.A, 46.1125°N, 65.6075°W, 29.VI–7.VII.2011, 7–13.VII.2011, 13–20.VII.2011, 20.VII–4.VIII.2011, 4–18.VIII.2011, M. Roy & V. Webster, old red oak forest, Lindgren funnel traps in forest canopy (16, AFC, NBM, RWC); Grand Lake Meadows P.N.A., 45.8227°N, 66.1209°W, 5–19.VII.2011, M. Roy & V. Webster, old silver maple forest with green ash and seasonally flooded marsh, Lindgren funnel traps in forest canopy (2, NBM, RWC). **York Co.**,15 km W of Tracy off Rt. 645, 45.6848°N, 66.8821°W, 30.VI–13.VII.2010, R. Webster & K. Burgess, old red pine forest, Lindgren funnel trap (in forest canopy) (1, RWC).

##### Collection and habitat data.

One adult of this species was collected from foliage of silver maple near a river. All others (19 specimens) from New Brunswick were captured in Lindgren funnel traps deployed in the canopy of an old red oak forest, an old silver maple forest, and an old red pine forest. No adults were captured in traps deployed near the forest floor at the above sites, indicating that this species may be most active in the forest canopy. Adults were captured during June, July, and August.

##### Distribution in Canada and Alaska

**.** ON, QC, NB (Bousquet 1991).

**Map 21.**
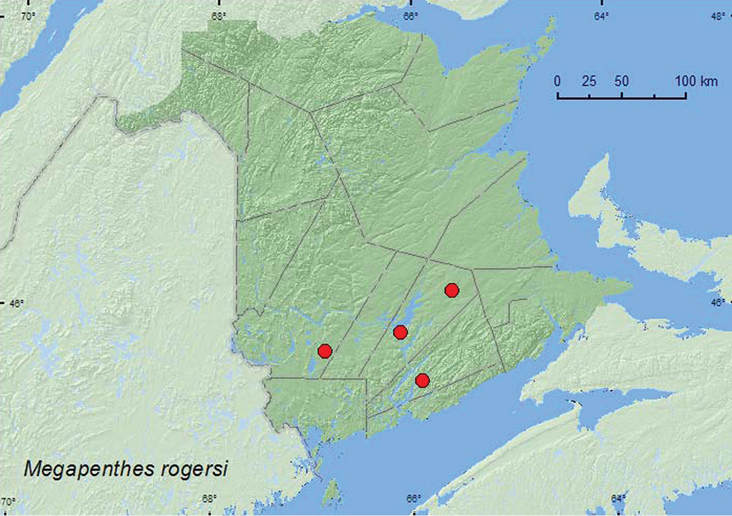
Collection localities in New Brunswick, Canada of *Megapenthes rogersi.*

#### Megapenthes solitarius

Fall, 1934**

http://species-id.net/wiki/Megapenthes_solitarius

Map 22

##### Material examined. 

**New Brunswick, Queens Co.**, Cranberry Lake P.N.A, 46.1125°N, 65.6075°W, 21–27.V.2009, 5–11.VI.2009, R. Webster & M.-A. Giguère, old red oak forest, Lindgren funnel traps (3, NBM, RWC); same locality data and forest type, 13–25.V.2011, 25.V–7.VI.2011, 22–29.VI.2011, M. Roy & V. Webster, Lindgren funnel traps (3, RWC). **Restigouche, Co.**, Dionne Brook P.N.A., 47.9064°N, 68.3441°W, 30.V-15.VI.2011, 27.VI–14.VII.2011, M. Roy & V. Webster, old-growth northern hardwood forest, Lindgren funnel traps (3, NBM, CNC).

##### Collection and habitat data.

In Alberta, two adults of *Megapenthes solitarius* were collected in mixed boreal forests; one from a window trap, the other was flying in a forest when captured (Fuller 2008). In New Brunswick, adults (9 specimens) of this rare species were captured in Lindgren funnel traps in an old red oak forest and an old-growth northern hardwood forest with sugar maple and yellow birch (*Betula alleghaniensis* Britt.). Adults were captured during May, June, and July.

##### Distribution in Canada and Alaska.

AB, QC, **NB** (Bousquet 1991; Fuller 2008).

**Map 22.**
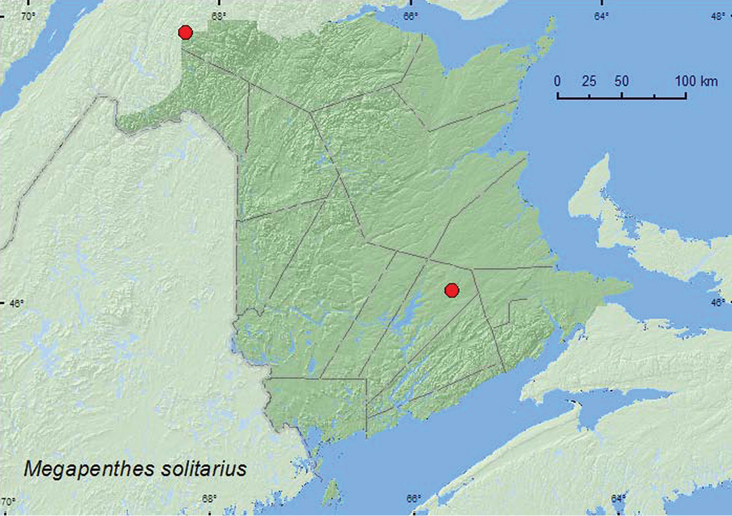
Collection localities in New Brunswick, Canada of *Megapenthes solitarius.*

### Tribe Melontini Candéze, 1859

#### Melanotus leonardi

(LeConte, 1853)**

http://species-id.net/wiki/Melanotus_leonardi

Map 23

##### Material examined.

**New Brunswick, Saint John Co.**, Saint John, Taylor’s Island 12.VI.1999, R. P. Webster, sea beach, under seaweed (1, RWC).

##### Collection and habitat data.

One adult was collected from under seaweed (drift material) on a sea beach during June along with many other Coleoptera species from other families.

##### Distribution in Canada and Alaska.

MB, ON, QC, **NB** (Bousquet 1991).

**Map 23.**
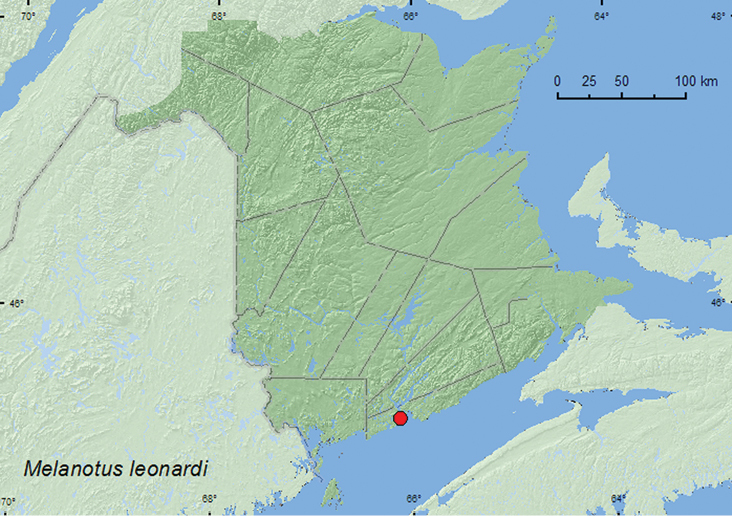
Collection localities in New Brunswick, Canada of *Melanotus leonardi.*

#### Melanotus sagittarius

(LeConte, 1853)**

http://species-id.net/wiki/Melanotus_sagittarius

Map 24

##### Material examined.

**New Brunswick, York Co.**, Charters Settlement, 45.8395°N, 66.7391°W, 27.VI.2006, 20.VII.2006, 10.VI.2007, 25.VI.2009, R. P. Webster, mixed forest, u.v. light (6, NBM, RWC).

##### Collection and habitat data.

Adults from New Brunswick were collected at an ultraviolet light in a mixed forest during June and July.

##### Distribution in Canada and Alaska.

QC, **NB** (Bousquet 1991).

**Map 24.**
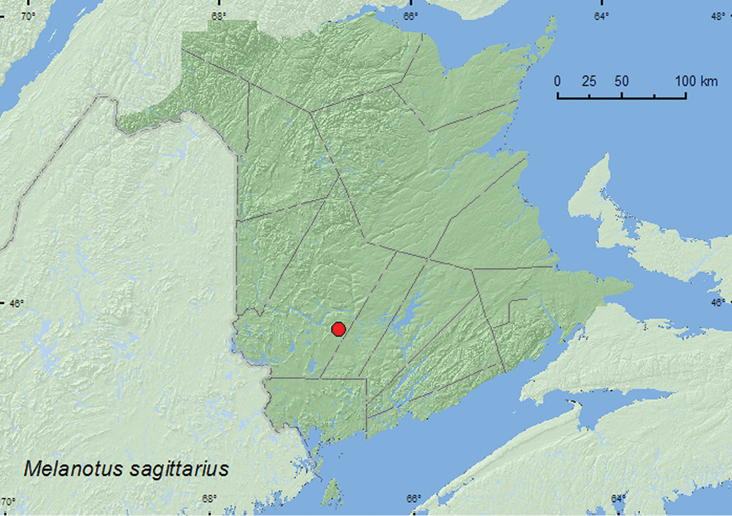
Collection localities in New Brunswick, Canada of *Melanotus sagittarius*.

## Supplementary Material

XML Treatment for Danosoma obtectum

XML Treatment for Athous posticus

XML Treatment for Athous scapularis

XML Treatment for Elathous discalceatus

XML Treatment for Hemicrepidius memnonius

XML Treatment for Hypoganus sulcicollis

XML Treatment for Hypoganus rotundicollis

XML Treatment for Oxygonus obesus

XML Treatment for Ligmargus lecontei

XML Treatment for Negastrius atrosus

XML Treatment for Paradonus pectoralis

XML Treatment for Agriotes quebecensis

XML Treatment for Agriotes pubescens

XML Treatment for Dalopius brevicornis

XML Treatment for Ampedus areolatus

XML Treatment for Ampedus nigricollis

XML Treatment for Ampedus oblessus

XML Treatment for Ampedus protervus

XML Treatment for Elater abruptus

XML Treatment for Sericus viridanus

XML Treatment for Megapenthes rogersi

XML Treatment for Megapenthes solitarius

XML Treatment for Melanotus leonardi

XML Treatment for Melanotus sagittarius
